# Editorial: Evolutionary adaptation in human-infecting fungi: ecological traits and pathogenicity

**DOI:** 10.3389/fcimb.2026.1820822

**Published:** 2026-03-24

**Authors:** Sara Mina, Nicolas Papon, Regina Helena Pires

**Affiliations:** 1 Department of Medical Laboratory Technology, Faculty of Health Sciences, Beirut Arab University, Beirut, Lebanon; 2 Univ Angers, Univ Brest, Fungal Respiratory Infections Research Unit, Federative Structure for Research in Health Sciences (SFR ICAT), Angers, France; 3 Laboratory of Microbiology, Universidade de Franca, Franca, Brazil

**Keywords:** adaptation, ecology, evolution, fungi, pathogenicity

The kingdom of fungi comprises a wide variety of organisms, adapted to an extensive range of ecological niches, including soil saprotrophs, plant symbionts, airborne species, and macromycetes. Interestingly, pathogenic fungi often share key ecological traits that are instrumental to infection, such as osmotolerance, melanization, thermotolerance, and morphogenetic switching (Corrêa-Junior et al., 2025). These traits have evolved independently across various fungal phyla, suggesting a common selective advantage in pathogenicity. Furthermore, extensive genetic diversity and dynamic regulatory networks promote the emergence of evolutionarily adaptable fungi capable of breaching the mammalian thermal barrier, establishing infection, and persisting under antifungal interventions (Brown, 2023).

Environmental disturbances like pollution, climate change, and anthropogenic activities, can alter soil ecosystems and may promote the development of new or more virulent fungal pathogens. This can expand the interface between humans and environmental fungi (Yiallouris et al., 2024). For example, soil-dwelling fungi like *Scedosporium*, *Aspergillus*, and *Fusarium* can withstand both natural and host-derived stresses thanks to their plastic genomes (Naranjo-Ortiz and Gabaldón, 2019). In addition, aquatic pathogenic fungi have acquired adaptive traits to cope with the ecological pressures within aquatic ecosystems (Weldon, 2025). This underscores the importance of investigating fungal ecology to gain a better insight into early risk assessments through continuous monitoring of environmental reservoirs as underscored by the One Health concept.

This Research Topic brings together a diverse collection of nine studies addressing the ecology, evolution, and pathogenic potential of human-infecting fungi across clinical, environmental, and geographic contexts. Collectively, these contributions highlight how fungal adaptation to distinct ecological niches shapes transmission routes, host–pathogen interactions, and disease outcomes.

First, Mina et al. investigated the distribution and ecological preferences of *Scedosporium* species in northern Lebanon. They show a predominance of *S. apiospermum* in soil samples and demonstrate that fungal presence was associated with soil parameters including pH, nitrogen, phosphorus, and organic matter content. They further performed genotyping analysis of the isolates, alongside foreign clinical isolates, using MultiLocus Sequence Typing (MLST) to characterize the population structure and highlight geographical clustering. Thus, it is particularly intriguing to identify the origin of this species using extended MLST genotyping in other geographic regions.

Additional investigations in this Research Topic provide insights into the characterization of aquatic yeasts. Fernández-Bravo et al. suggest that *Phialophora submerse*, a black yeast species isolated from freshwater sediments in Spain, exhibits distinct virulence profiles and stress tolerance mechanisms when compared to *P. verrucosa* and *P. americana*. This may be attributed to their ecological origins and pathogenic capabilities, suggesting species-specific pathogenicity of *Phialophora*. In addition, Oliveira et al. report, for the first time, the detection of *Candida palmioleophila* in a commercially relevant fish species belonging to the Sciaenidae family (Cynoscion sp.) using Matrix-Assisted Laser Desorption/Ionization Time-of-Flight Mass Spectrometry (MALDI-TOF MS). This study opens new windows for future assessment of potential risks associated with this emerging pathogen.

Clinical and disease-oriented aspects are addressed through investigations into tinea capitis, fungal keratitis, and cryptococcal infections. In particular, a prospective study conducted by Zheng et al. in the tropical province of Hainan, China, analysed the epidemiology and pathogenic spectrum of scalp dermatophytes between 2023 and 2024, revealing a predominance of cases in children under 10 years of age and a strong association with contact with domestic animals. The zoophilic species *Microsporum canis* emerged as the main etiological agent, with highly inflammatory lesions (kerions) representing the most frequent clinical presentation. This study highlights the relevance of dermoscopy for accurate diagnosis and reports a shift in dominant pathogens linked to increasing pet ownership, while confirming the high efficacy of systemic oral antifungal therapy in achieving clinical and mycological resolution.

Further contributions within this Research Topic highlight the pathogenicity of *Cryptococcus*. Cryptococcal disease is explored from complementary clinical, ecological, and molecular perspectives. A longitudinal clinical study by Su et al. involving HIV-infected patients with cryptococcal meningitis monitored cytokine profiles in blood and cerebrospinal fluid during antifungal therapy, revealing a marked reduction in inflammatory mediators over 28 days and indicating a progressive restoration of immune homeostasis. Importantly, this study demonstrated that immune responses within the central nervous system differ substantially from systemic responses, underscoring the complexity of host–pathogen interactions and highlighting the need for personalised immunomodulatory strategies to reduce the high mortality associated with this condition. From an ecological and evolutionary standpoint, Irere et al. propose that environmental niches precondition *Cryptococcus neoformans* for distinct disease trajectories prior to human infection. Nutrient-rich habitats such as pigeon guano, abundant in phosphate, favour systemic dissemination, whereas inositol-rich eucalyptus trees selectively prime the fungus for neuroinvasion. This ecological imprinting provides a compelling explanation for the disproportionately high burden of severe cryptococcal meningitis observed in eucalyptus-rich regions, particularly in parts of Africa and Asia, and emphasises the importance of environmental surveillance in the epidemiology of fungal diseases.

At the molecular level, Timboni et al. investigated the role of phosphatidylcholine biosynthesis pathways in *C. neoformans* pathogenicity. Disruption of key metabolic routes resulted in impaired membrane stability, reduced stress tolerance, and loss of major virulence traits, leading to pronounced hypovirulence and a markedly diminished capacity to colonise the central nervous system *in vivo*. These findings highlight metabolic flexibility as a critical determinant of fungal survival and dissemination within the host and identify the glycerophosphocholine reacylation pathway, which is absent in mammals, as a promising target for the development of selective antifungal therapies.

Finally, this Research Topic ends with a deeper understanding of host-pathogen interactions, particularly focusing on *Candida albicans*, *Fusarium* spp., and *Aspergillus flavus*. Blázquez-Muñoz et al. demonstrate that the chitinase Cht3 plays a crucial role in maintaining cell surface architecture and contributes to the virulence of *Candida albicans*. Dinesh et al. identify specific differentially expressed genes (DEGs) mediating both pathogen-specific and pathogen-independent mechanisms in fungal keratitis caused by *Fusarium* spp. and *Aspergillus flavus*.

Overall, this Research Topic illustrates the breadth of contemporary mycology research, integrating environmental surveillance, clinical investigation, and molecular approaches to enhance our understanding of fungal pathogenicity and adaptation. By bridging ecological traits with clinical relevance, the collected works provide valuable insights into the evolving landscape of human-infecting fungi and provide a foundation for future research and public health strategies.
